# Poststroke Depression, An Underrated Clinical Dilemma: 2022

**DOI:** 10.7759/cureus.32948

**Published:** 2022-12-26

**Authors:** Aneeque Jamil, Denise Csendes, Sai Dheeraj Gutlapalli, Keerthana Prakash, Kiran Maee Swarnakari, Meena Bai, Mohana Priya Manoharan, Rabab Raja, Aditya Desai, Darshi M Desai, Ana P Arcia Franchini

**Affiliations:** 1 Internal Medicine, California Institute of Behavioral Neurosciences & Psychology, Fairfield, USA; 2 Medicine, California Institute of Behavioral Neurosciences & Psychology, Fairfield, USA; 3 Internal Medicine Clinical Research, California Institute of Behavioral Neurosciences & Psychology, Fairfield, USA; 4 Internal Medicine, University of California Riverside School of Medicine, Riverside, USA; 5 Research, California Institute of Behavioral Neurosciences & Psychology, Fairfield, USA

**Keywords:** poststroke depression, stroke and mental health, stroke and psychiatry, depression, stroke

## Abstract

It is known that the majority of patients are prone to develop depression following a stroke. Several biological factors, including the disruption of the hypothalamic and adrenal axis and changes in cortisol and interleukin 6 (IL6), are said to have an essential role in its development. Magnetic resonance imaging (MRI) scans point toward white matter lesions and lacunar infarcts as the primary pathological culprit. People affected by poststroke depression (PSD) are more likely to commit suicide or develop another ischemic event after the initial episode, which can likely increase the mortality related to PSD and stroke. Selective serotonin reuptake inhibitors (SSRIs) are the mainstay of treatment for PSD. However, it has a poor safety profile and is not very productive, making the use of SSRIs controversial, and further studies are required to prove its benefits concerning PSD. This literature review discusses the importance of PSD, how it impacts the quality of life of people affected by stroke, and its treatment.

## Introduction and background

Cerebrovascular accident (CVA), also known as stroke, has been an overall burden to society. According to the statistical data of the European Union, in 2017, 1.12 million stroke cases were recorded, of which there were 9.6 million survivors and 0.46 million deaths. It is estimated that over the next 30 years, there will be a 27% increase in stroke patients in the European Union [1]. Stroke is not only a problem for stroke victims but also a factor causing distress to family members. Patients can become paralyzed and dependent on their family members for nursing and general care. This loss of autonomy leads to a psychological element of stroke, often neglected. It is estimated that 33.3% of the patients affected by stroke eventually develop poststroke depression (PSD) [2]. PSD is divided into two phases - early and late. The most common phase, the early stage, develops within three months of the initial episode, and the late phase develops after three months. Symptoms most often experienced are similar to those usually seen in major depressive disorder; however, sleep disturbances and social withdrawal are more pronounced with PSD [2].

Studies have shown that depression is the most common psychiatric complication following CVA [3]. The pathogenesis of PSD has been attributed to various factors, including changes in the hypothalamic-pituitary-ovarian (HPO) axis and monoamine neurotransmitters. Moreover, genetics, immunological factors, and psychosocial aspects contribute to its development [3]. Data from several autopsies have shown some histological evidence that lacunar infarcts and white matter lesions are significant contributors to depression, memory loss, and poor executive functions in the elderly experiencing PSD [4]. Nonetheless, the role of magnetic resonance imaging (MRI) changes in the brain and their association with depression is still unclear [4].

It seems as if more studies have been conducted on the physical outcomes of CVA, which is undoubtedly the main component of the disease, as the physical aspects are the seed of other complications that may follow. However, mental aspects of the disease are one of the significant complications, which are often overlooked and need to be discussed in more detail. It is known that people affected by PSD are more likely to have another episode of stroke after the initial attack [3]. The intensity of PSD is such that it can lead patients to the extent of having active suicidal ideations and attempts [5].

As the recurrence of CVA is associated with depression and increased mortality [3], it is essential to consider the psychological aspects of CVA and be given equal importance to its physical components, for this will positively affect the prognosis of the disease. Therefore, this literature review will focus on what causes PSD to understand better its pathophysiology, its consequences, and why it is essential to raise awareness about this issue. Furthermore, we will discuss the different factors that affect this association and the challenges we face today with the treatment of PSD. It is essential to bring up the discussion so its awareness might help appropriate screening tools for identifying PSD following stroke and formulate proper therapeutic practices - to help reduce recurrent ischemic attacks and suicidal attempts due to PSD.

## Review

Discussion

This section sheds more light on the various aspects of PSD, including its etiology, consequences, treatment, and early detection of its development. Studies will further validate the significance and loopholes of every aspect and what is known and unknown. 

Pathophysiology

The pathophysiology of PSD is divided into two components, biological and psychosocial factors. If we discuss the physical aspects, inflammatory cytokine interleukin-6 (IL6) and cortisol have been linked to PSD. It is thought that after a stroke, there is a disruption in the hypothalamus-adrenal axis, which results in PSD. A study was conducted by Tongji Hospital from July 2014 to December 2015. A total of 100 patients were taken and divided equally into controls and subjects (50 each). One week following the stroke, the serum levels of cortisol and IL-6 were taken. IL6 levels were measured by enzyme-linked immunosorbent assay and cortisol levels through chemiluminescence immunoassay. Three weeks following the stroke, the Diagnostic and Statistical Manual of Mental Disorders (DSM1V) and Hamilton Depression Rating scale (Hamd-21, score > 7) were used to create two groups, one who had PSD and the other without PSD [6]. This study showed a significant increase in cortisol and IL6 in the PSD group compared with the non-PSD group. Cortisol and IL6 increase the risk of PSD, and there may also be a temporal relationship between them. More studies should be conducted to evaluate how PSD is affected by time with respect to IL6 and cortisol and whether other comorbidities affect this association. Nonetheless, high levels of cortisol in blood have been linked to depression in general, leading us to another question: Is cortisol a marker of depression [7,8]?

There is another way in which the location of the infarct can help us find the cause of PSD. White matter lesions and lacunar infarct are related to PSD [4]. To see this association, a study was started in 2013, and 374 patients were selected by 2016; the study's goal was to check if cerebral small vessel disease (CSVD) in patients with lacunar infarct was related to PSD. An ordinal score of 0-4 was calculated by considering lacunar infarcts, cerebral microbleeds, perivascular spaces, and white matter lesions, and the development of PSD was analyzed. It showed that 24.1% of the patients with a high vascular burden who had lacunar infarcts developed PSD [9]. To further validate the association between white matter lesions, lacunar infarcts, and periventricular infarcts, another small cohort study was conducted in 2013, which showed no association between these lesions and the development of depression [4]. The relationship between MRI lesions and the development of depression remains controversial. The recent study, depicted in the text above, conducted in 2016 shows some association between lacunar infarcts and white matter lesions with PSD. Therefore, it would not be wrong to say that this association needs more clarity with a large sample size to conclude; however, white matter lesions and lacunar infarcts are the closest MRI lesions related to PSD.

Besides the biological factors, psychosocial aspects also act as a catalyst in aggravating depression in patients following stroke. To check this association, a longitudinal study was conducted on 125 patients. This study assessed the patients with relationship problems and limited activity following stroke through Cornell Scale for Depression (CSD) and Montgomery and Åsberg Depression Rating Scale (MARDS). It was seen that people who had relationship problems had more than 4.4 times higher risk of developing PSD and those with limited activity also had a higher probability of being affected by PSD following stroke [10]. People who go into social isolation and have functional impairment fall under the DSM1V category for depression. Moreover, in stroke, people are paralyzed, creating an additional risk factor for depression, and hence are more likely to develop PSD.

**Figure 1:**
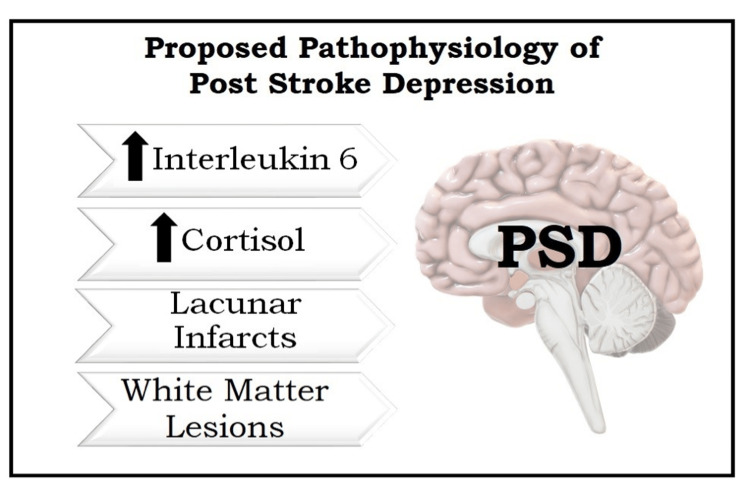
Proposed pathophysiology of PSD (upward arrow: increased). Figure credits: Aneeque Jamil PSD, poststroke depression

It seems studies conducted so far have only shown some associations about the pathophysiology of PSD without identifying any specific etiology, and no fixed model, criteria, or lab workup has been set to point toward a specific etiology of PSD. Further studies should be carried out to clearly understand what is causing the underlying psychiatric disability following a stroke. The pathophysiology of PSD is summarized in Figure 1.

PSD and Suicide

Eighty percent of the primary studies show that the risk of suicide increases following stroke [11]. According to one study, the risk of suicide among stroke patients is twice as high compared to the general population [12]. To check how strongly stroke and suicide are linked, in 2013, a population-based study was conducted in Korea. This study collected data from 228,735 individuals, of whom 4,560 patients were affected by a stroke. Patients were adjusted for socioeconomic, demographic, physical, and mental health to avoid confounders. It was seen that people who had a stroke were more likely to develop suicidal ideations and were more likely to complete suicide attempts [5]; thus, stroke alone may have contributed to suicide in patients with PSD. To further check if suicide in stroke patients was affected by other factors, a study was carried out in 2013 at Hua Hei Hospital in China.

A total of 271 patients took part in the study, out of which only 29 patients were diagnosed with having suicidal ideations through the Beck Scale for Suicide Ideation (BSI). It was seen that those 29 patients who had suicidal ideations had PSD, symptoms of fatigue, and displayed physical activity, coping ability and belief in the treatment. All 29 patients were found to be statistically significant on logistic regression analysis [13], showing that these factors were present in those 29 patients. This led to the precipitating factors discussed earlier also being associated with suicide in stroke patients. It can be inferred that things that negatively impact our mind and body act as a catalyst in developing suicidal ideations in patients affected by PSD.

**Figure 2:**
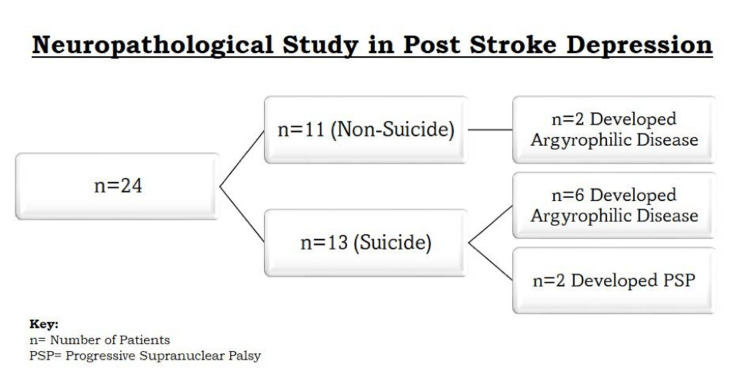
Neuropathological study in PSD. Figure credits: Aneeque Jamil n, number of patients; PSP, progressive supranuclear palsy; PSD, poststroke depression

Another study showed that 15% of people affected by PSD are reported to have suicidal ideations and eventually commit suicide [14]. Moreover, the chances of having suicidal ideations were twice as in patients with PSD [14]. A retrospective study was conducted to check the association between pathological effects on neurons caused by various diseases and its impact on suicide related to PSD. A total of 1042 medico-legal autopsies were taken between 2006 and 2013. Patients were assessed and diagnosed with PSD with appropriate tools and specialists to avoid misclassification. Furthermore, data regarding suicide was collected through family interviews, hospital records, and autopsy findings. The controls were patients with PSD whose cause of death was other than PSD. It was seen that 24 patients could finish the study, of which 11 died of suicide and 13 died from causes other than suicide. Argyrophilic grain disease was found in 6 out of 11 patients who were affected by suicide and 2 out of 13 nonsuicidal patients, and the results were statistically significant, which was similar to the case with progressive supranuclear palsy in which two patients in the suicide group were found to have the disease [14]. As the results were statistically significant for both disorders with suicidal patients, the disorders may have contributed to the development of suicide ideation in patients with PSD. However, if the sample size used was large, it would have given a better picture of the association, but this seems a limitation where autopsies were used to conclude a study. The study results are shown in Figure 2.

PSD and Risk of Recurrent Stroke

The linkage between the recurrence of stroke and PSD is still debatable [3]. To check whether PSD is linked to stroke recurrence, a study was conducted in which data was pooled from several studies; in the end, six studies on 4,648 patients were found to have a relative risk of 1.48 when adjusted for the recurrence of stroke in a fixed-effect model. When subgroup analyses were conducted, the chance of re-ischemia was higher in people affected by PSD compared to other subgroups of patients affected by stroke [3]. PSD may be a risk factor for recurrent ischemia. To further verify the relationship, another cohort study was conducted. The effect of PSD and depression-executive dysfunction syndrome (DES) was carried out, and its impact on recurrent ischemia was analyzed. In this study, 486 cohorts were taken from Helsinki Hospital, of which 223 were followed for 12 years. Inclusion criteria included patients who had their first stroke episode and were able to be tested for psychological, executive, and neurological assessments. The results showed that patients with PSD were more likely to have another ischemic episode, and patients with DES were at a greater risk of recurrent ischemia than PSD [15]. It is well known that depression increases the risk of stroke [1], and PSD increases the mortality rate in stroke survivors [16]. An increase in mortality might be due to recurrent ischemia, and those with poor executive function are at a greater risk. There should be more data to conclude this association so that appropriate treatment is directed at the right time, which can help reduce the mortality related to PSD and stroke.

PSD and Its Treatment

This section focuses on different medications used for PSD and their effects on it. Currently, selective serotonin reuptake inhibitors (SSRIs) are the primary treatment for PSD. A randomized controlled trial was carried out in 35 rehabilitation centers in Sweden. The study aimed to see the effects of fluoxetine on acute stroke and included 1,500 patients aged 18 or more. The patients were equally divided into two groups; one of the groups received 20 mg of fluoxetine, and the control group received a placebo. The patients were followed up after six months using the modified rank scale (MRS). The study results showed that although there was no improvement in stroke after six months, fluoxetine showed improvements in acute depression. The downside of using fluoxetine was that it caused adverse effects such as hyponatremia [17,18]. Another study on the effects of paroxetine showed no benefit when used for PSD, although it had no adverse effects compared to fluoxetine [19]. The use of fluoxetine for PSD is a double-edged sword. It is favorable for treating acute episodes of depression compared to paroxetine, but it has some side effects. A follow-up study was conducted on acute stroke patients to check the antiplatelet effect of SSRI on patients affected by acute stroke. In this study, data was gathered from Danish medical registries, 5,583 SSRI users were taken from the data and were matched using propensity scoring, Cox regression was used to calculate the hazard ratio, and the follow-up time was 1,159 days. The results showed that the risk of recurrent stroke and cardiovascular complications decreased and were statistically significant in SSRI users; however, the risk of major bleeding, intracranial bleeding, mortality rate, and death caused by bleeding increased, of which major bleeding was statistically significant (Table 1) [20,21].

**Table 1:** Adverse effects of SSRIs. SSRI, selective serotonin reuptake inhibitor

Effects of SSRI on acute stroke patients	Hazard ratio	95% confidence interval
Myocardial infarction and recurrent stroke	0.77	0.62-0.96
Major bleeding	1.33	1.14-1.55
Intracranial bleeding	1.14	0.62-2.12
Mortality among SSRI users	1.13	1.00-1.28
Death caused by bleeding	1.89	0.97-3.66

These statistics still make SSRIs controversial when their complications are weighed against the benefits. This indicates that helping stroke patients with mood swings might lead to another irreversible pathologic cause, that is, major bleeding.

Cognitive behavioral therapy (CBT) is a treatment option other than SSRIs, which can be used to check its effectiveness.

A meta-analysis of randomized controlled trial was conducted using a variety of databases. A total of 23 studies were conducted. The results of the studies showed improvements in the mental health and overall mood of the patients. These statistically significant positive results were considered for patients who were treated with both CBT and antidepressants and CBT alone. The results depended on the basal depression status, age of the patient, and duration for which the CBT was used [22]. Another study showed positive results with CBT, which included stroke survivors affected by depression and people taking care of them [23]. Apart from its positive effects on PSD, CBT has proven to be beneficial in people affected by poststroke fatigue and sleep disturbances [24]. CBT is known for the treatment of depression. In that context, the same could be true for PSD. Some studies have shown positive results of CBT concerning PSD. However, further experiments should be conducted to have a greater insight into how effective it is for the treatment of PSD.

From the aforementioned discussion, all the treatment modalities used for PSD have some pros and cons. The consequences of using SSRIs can be unpleasant. Hence, its usage today depends on the cost-benefit analysis along with the clinician's sense of considering the situation of the patients, as its use remains controversial. Studies should be carried out to determine the role of CBT as it is a nonpharmacological intervention without side effects. Unfortunately, fewer studies have been performed to date on CBT. It should be studied in more detail to establish a promising treatment modality for PSD. The treatment summary of PSD is given in Table 2.

**Table 2:** Treatment summary of PSD. PSD, poststroke depression

Treatment	Efficacy	Side effects
Fluoxetine	Good	Poor
Paroxetine	Poor	No side effects
Cognitive behavioral therapy	Good (insufficient data)	No side effects

*Limitations* 

This study could have had more specific limitations when it comes to age and gender, as the age group was not specified and males and females were included in the study. Some studies had a smaller sample size. These factors broadened the review and might have impacted its outcome and specificity.

## Conclusions

PSD is an issue that is not given the importance that it deserves. As discussed in this paper, the consequences of PSD are not limited only to the patient's mental well-being. It could also worsen the prognosis of stroke due to recurrent ischemic attacks. PSD-related suicide also contributes to the stroke-related mortality rate. Appropriate screening tools with higher sensitivity should be devised for their early detection. Pathophysiology of PSD remains hazy as no single mechanism of action is found to be the cause of symptoms. Further research should be conducted to elucidate the pathophysiology so that we could establish the appropriate treatment accordingly. The treatment of PSD has some issues with the side effects of SSRI, such as major bleeding. So its use should be consulted in people who are on anticoagulants or are affected by other problems that may result in harmful consequences. This leads us to nonpharmacological interventions such as CBT, which should be further studied as their effectiveness comes at the cost of no side effects. If studies can gather better results and a clear picture of the *missing tiles* related to PSD, it will help reduce the mortality associated with PSD. Overall, PSD is an underrated issue that should be given importance and awareness, as its appropriate management can create a difference among stroke survivors or at least can improve the quality of their life.
